# Analysis of relevant factors influencing size of breast abscess cavity during lactation: a cross-sectional study

**DOI:** 10.1186/s40001-024-01733-7

**Published:** 2024-02-20

**Authors:** Gao Yajun, Zou Yan, Zhang Yi, Chen si, Li yan, Ding Songtao

**Affiliations:** 1 Department of Breast Surgery, Haidian District Maternal and Child Health Hospital, Beijing, China; 2 National Research Institute for Family Planning, Beijing, China

**Keywords:** Breastfeeding, Abscess, Non-medical massage, Antibiotic

## Abstract

**Objective:**

The aim of this study was to investigate risk factors for the severity of breast abscess during lactation.

**Methods:**

A cross-sectional study was conducted using data from the Questionnaire survey of breast abscess patients. According to whether the maximum abscess diameter > 5 cm, the patients were divided into two groups for univariate and multivariate regression analysis.

**Results:**

1805 valid questionnaires were included. Univariate and Binary logistic regression analysis demonstrated that low education (OR = 1.5, 95% CI 1.1–2.0, *P* = 0.005), non-exclusive breastfeeding (OR = 0.7, 95% CI 0.6–0.9, *P* = 0.004), fever > 37.5 ℃ (OR = 0.7, 95% CI 0.6–0.9, *P* = 0.003), flat or inverted nipples (OR = 0.7, 95% CI 0.6–0.9, *P* = 0.005), antibiotic used (OR = 0.7, 95% CI 0.6–0.9, *P* = 0.006), and non-medical massage (OR = 0.3, 95% CI 0.2–0.4, *P* < 0.001) were the effective independent influencing factors for the maximum breast abscess diameter > 5 cm.

**Conclusion:**

Low education, non-exclusive breastfeeding, fever > 37.5 ℃, inverted or flat nipples, antibiotic used, and non-medical massage history have adverse effects on the severity of breast abscess during lactation.

## Introduction

Breastfeeding baby to the age of 2 years or above is the best feeding mode recommended by the World Health Organization (WHO) in 2009. However, it is reported that the incidence of mastitis during lactation is 2–33% [1], which will inevitably affect breastfeeding [2]. Breast abscess during lactation is the most common severe complication of mastitis [3], Approximately 3–11% of women with acute mastitis will develop an abscess [4]. The overall incidence of breast abscess in lactating women is low, reported as 0.1–3% [5]. Breast abscess is traditionally treated by incision and drainage. This treatment is invasive and needs repeated dressing changes in the later stage, with scars left and a high delactation rate. Giess et al. [6] suggested that some breast abscesses during pregnancy and lactation can be treated by puncture and pus aspiration. For breast abscesses with a maximum diameter   5 cm, the clinical studies recommend minimally invasive puncture and pus aspiration, which is minimally invasive technique with low delactation rate [7–9]. The size of breast abscess is closely related to treatment modality and prognosis [7, 8]. As early as possible finding lactating breast abscess and judging its size is important for timely and personalized treatment. Therefore, through this multicenter cross-sectional study, we aim to explore the relevant factors influencing the size of the breast abscess cavity of the patients during lactation, so as to reduce the occurrence of incision and drainage for large abscesses during lactation, thus reducing the rate of weaning.

## Methods

### Study participants

In this study, the lactating patients with initially diagnosed breast abscesses in the Department of Breast Surgery of 24 hospitals in 15 provinces, cities and regions in China, from October 2019 to June 2021 were selected as subjects. Before treatment, an online questionnaire survey was conducted on the Golden Data platform through scanning code assisted by the trained medical personnel. The questionnaire was self-designed and was verified by experts. The details include the basic information, family economic level, feeding situation, mastitis condition, massage history, previous treatment (including the use of antibiotics during breast inflammation), and breast ultrasound lesions were recorded through questionnaires. No less than 50 patients of breast abscesses during lactation every year were required for the units participating in this survey. All the patients participated in the survey had signed the informed consent online.

### Data collection and grouping

The baseline data, pregnancy and childbirth data, feeding mode, basic condition of disease, treatment after onset, and maximum breast abscess diameter measured by ultrasound were collected.

According to relevant guidelines and literature [7, 8] the maximum diameter of the breast abscess cavity during lactation > 5 cm will increase the probability of puncture failure, and catheterization or incision and drainage is recommended. Therefore, the patients in this study were divided into two groups: maximum breast abscess diameter ≤ 5 cm and maximum breast abscess diameter > 5 cm.

### Data analysis and statistical methods

The diagnostic criteria for breast abscesses during lactation were as follows: in the late stage of lactational mastitis, there was still a well-defined mass on the breast, which might be accompanied by redness, rigidity and tenderness, and there was a flowable fluid sonolucent area by ultrasonic examination [3].

The collected data in this study were descriptively analyzed by SPSS 25.0. Subsequently, univariate analysis was performed with the *χ*^2^ test (the variables with *P* < 0.05). In the binary logistic regression model for multivariate analysis, odds ratio (OR) and 95% confidence interval (CI) were calculated. Bilateral *P* < 0.05 was considered as statistically significant.

## Results

**Fig. 1:**
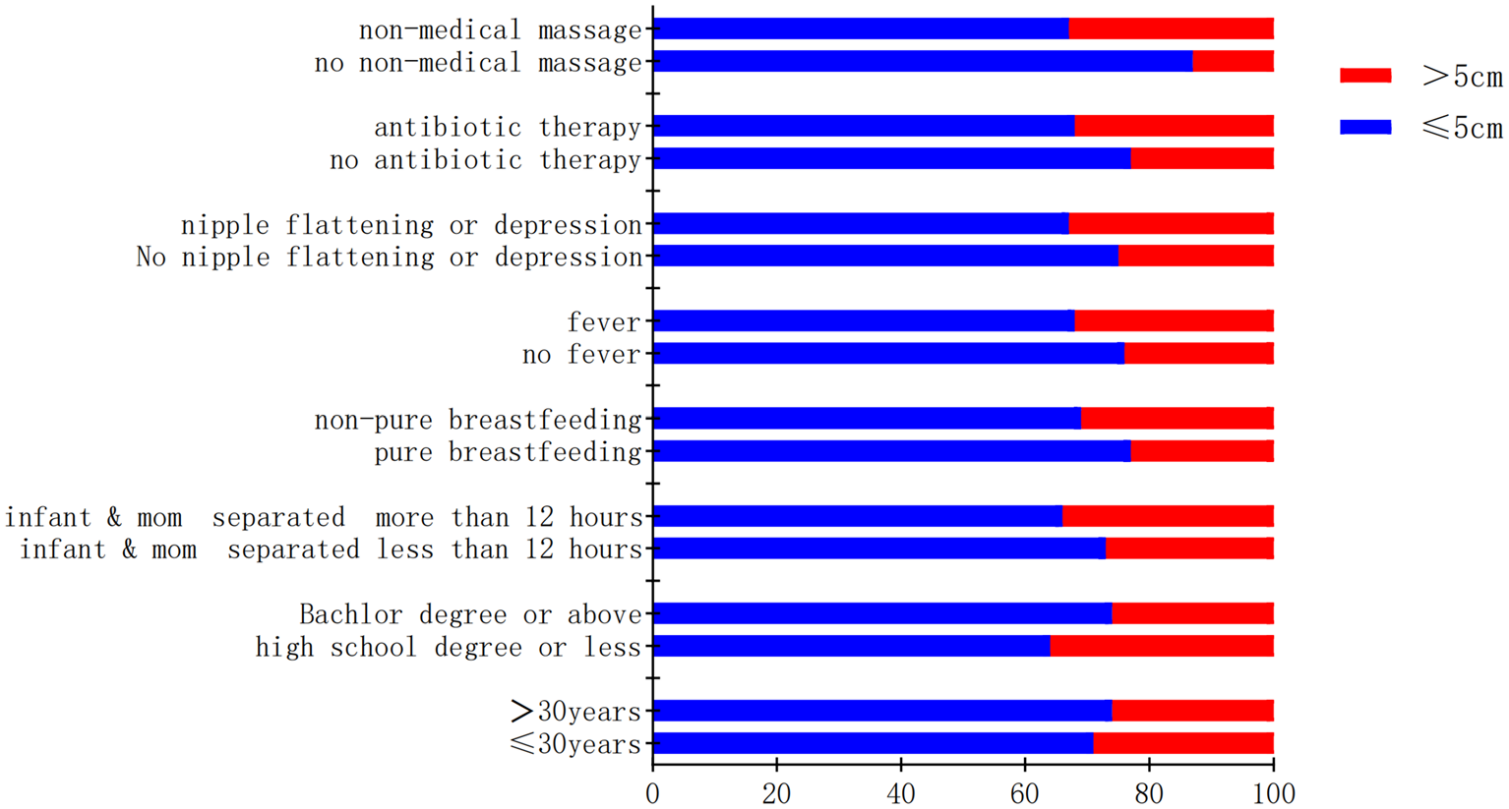
The proportion diagram of each factor in two groups

A total 1842 patients were investigated by questionnaires, and 1805 valid questionnaires were included for analyzed after excluding the samples with missing key information. Patients ranged in age from 18 to 50 years, with an average age of 30.36 years. Education: high school or below accounted for 15.5%, university degree accounted for 64%, and the rest accounted for 20.5%. Mode of production: 62.2% of the cases were vaginal delivery; cesarean section accounted for 37.8%. The first birth accounted for 83.1%; 16.2% had a second child; more than 2 births accounted for 0.7%. 91.4% of the patients had no history of diabetes. Among them, there were 1305 (72.3%) patients with the maximum breast abscess diameter ≤ 5 cm and 500 (27.7%) with the maximum breast abscess diameter > 5 cm. Univariate analysis showed that educational background, maternal–infant separation, feeding mode, body temperature, flat or inverted nipples, antibiotic used, and non-medical massage history of the patients were correlated with whether the maximum abscess diameter > 5 cm, but no correlations in other factors (Table 1, Fig. 1).

**Table 1:** Univariate logistic regression analysis

Variables	Maximum abscess (*n*)		> 5 cm, *n*%	Chi-square	*P* value
	≤ 5 cm	> 5 cm			
Age (years)					
≤ 30	689	285	29.26%	2.571	0.109
> 30	616	215	25.87%		
Firstborn					
No	211	94	30.82%	1.783	0.182
Yes	1094	406	27.07%		
Production pattern					
Eutocia	816	306	27.27%	0.271	0.602
Cesarean	489	194	28.40%		
Education high school and below					
Yes	180	100	35.71%	10.626	0.001
No	1125	400	26.23%		
Separation of infant and mom					
No	1185	438	26.99%	4.095	0.043
Yes	120	62	34.07%		
Pure breastfeeding					
Yes	578	171	22.83%	15.163	< 0.001
No	727	329	31.16%		
Fever > 37.5 ℃					
No	718	226	23.94%	13.971	< 0.001
Yes	587	274	31.82%		
Flat or sunken nipples					
No	930	315	25.30%	11.538	0.001
Yes	375	185	33.04%		
Used antibiotics					
No	671	202	23.14%	17.571	< 0.001
Yes	634	298	31.97%		
Non-medical massage					
No	425	66	13.44%	68.471	< 0.001
Yes	880	434	33.03%

**Fig. 2:**
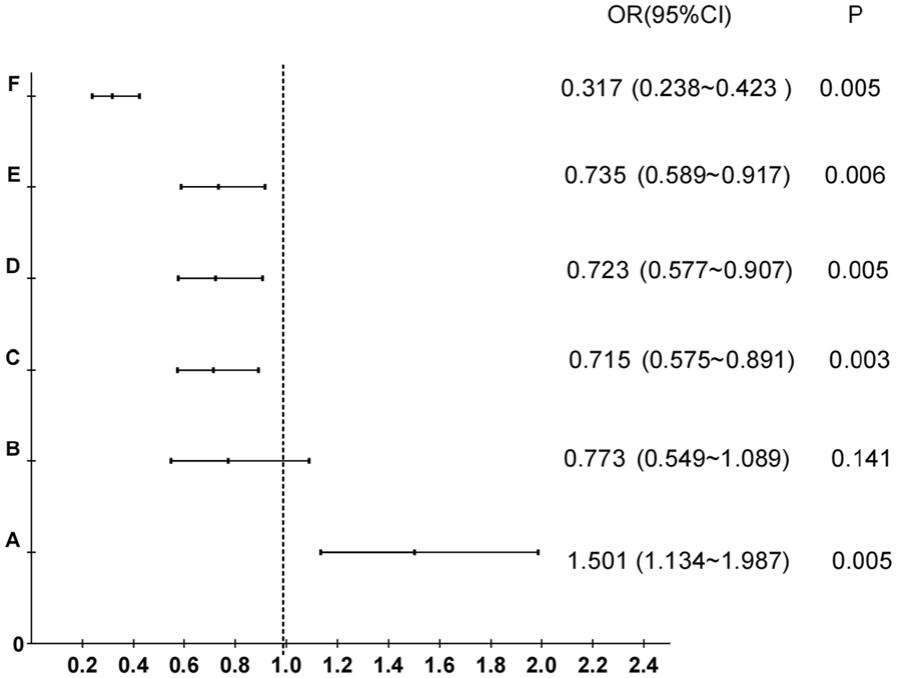
Factors influencing the maximum diameter of an abscess > 5 cm. **A** Educational: university undergraduate above as reference; **B** Breastfeeding: non-exclusive breastfeeding as reference; **C** Fever: > 37.5 ℃ as reference; **D** Inverted or flat nipples: inverted or flat nipples as reference; **E** Antibiotic: antibiotic used as reference; **F** breast massage: non-medical breast massage history as reference

Binary logistic regression analysis (Table 2) demonstrated that high school education and below (OR = 1.5, 95% CI 1.1–2.0, *P* = 0.005), non-exclusive breastfeeding (OR = 0.7, 95% CI 0.6–0.9, *P* = 0.004), history of fever > 37.5 ℃ (OR = 0.7, 95% CI 0.6–0.9, *P* = 0.003), flat or inverted nipples (OR = 0.7, 95% CI 0.6–0.9, *P* = 0.005), antibiotic use history (OR = 0.7, 95% CI 0.6–0.9, *P* = 0.006), and non-medical massage (OR = 0.3, 95% CI 0.2–0.4, *P* < 0.001) were the effective independent influencing factors for the maximum breast abscess diameter > 5 cm of the patients during lactation (Fig. 2).

**Table 2:** Binary logistic regression analysis

Variables	B	SE	Wald	df	*P* value	OR	OR 的 95% CI	
							Low	High
Education high school and below								
Yes	0.406	0.143	8.065	1	0.005	1.501	1.134	1.987
No	0					Reference		
Separation of infant and mom								
No	− 0.257	0.175	2.166	1	0.141	0.773	0.549	1.089
Yes	0					Reference		
Pure breastfeeding								
Yes	− 0.328	0.115	8.151	1	0.004	0.721	0.576	0.902
NO	0					Reference		
Fever > 37.5 ℃								
No	− 0.335	0.112	8.982	1	0.003	0.715	0.575	0.891
Yes	0					Reference		
Flat or sunken nipples								
No	− 0.324	0.116	7.861	1	0.005	0.723	0.577	0.907
Yes	0					Reference		
Used antibiotics								
No	− 0.308	0.113	7.461	1	0.006	0.735	0.589	0.917
Yes	0					Reference		
Non-medical massage								
No	− 1.148	0.146	61.498	1	< 0.001	0.317	0.238	0.423
Yes	0					Reference		
Constant	0.109	0.195	0.312	1	0.576	1.115		

CI: confidence interval

## Discussion

Relevant studies have found by bacterial culture of breast milk from patients with mastitis that staphylococcus aureus accounts for about 63%, which can produce toxins and thereby cause influenza-like symptoms such as high fever [10]. Our study showed that among the 1805 patients with breast abscesses during lactation, 861 had body temperature > 37.5 ℃, accounting for 47.7%, which shows that medium–high fever is not a necessary indicator to diagnose breast abscess. Under physiological conditions, coagulase-negative *Staphylococci* (CoNS) and viridans *Streptococci* (i.e., *S. mitis* and *S. salivarius*) form thin biofilms that line the epithelium of the mammary ducts, allowing a normal milk flow [11]. In the setting of dysbiosis, these species proliferate and function under opportunistic circumstances whereby they are able to form thick biofilms inside the ducts, inflaming the mammary epithelium and forcing milk to pass through an increasingly narrower lumen. CoNS and viridans Streptococci do not produce toxins responsible for acute bacterial mastitis; therefore, systemic symptoms are uncommon and local breast symptoms are milder than in acute mastitis.

CoNS and *Streptococcus viticulturae* are colonizing bacteria, accounting for about 33% of the bacterial cultures in milk from lactating mastitis and their overgrowth can lead to subacute or subclinical mastitis. In addition, they will form a thick biofilm in the duct, blocking the flow of breast milk and causing breast engorgement [12]. Local breast pain without breast redness and fever is prone to be ignored clinically, resulting in early delactation. Consequently, attention should be paid to painful breast masses without fever during lactation, and an ultrasound should be carried out to identify abscesses. The proportion of body temperature > 37.5 ℃ of the patients with maximum diameter > 5 cm is higher than those of the patients with abscess maximum diameter < 5 cm, which indicating that patients with a history of moderate or high fever have the increased chance of enlarged breast abscess, and the higher body temperature of the patients suggests the more severe infection [13].

This survey found that mothers with high level of education (university degree or above) had a significantly lower probability of breast abscess cavity > 5 cm. and the possibility of maximum abscess diameter > 5 cm of the patients with exclusive breastfeeding is 27.9% lower than that of the patients with mixed and artificial feeding. A previous study [14] have also confirmed that mothers with low educational background have a lower rate of exclusive breastfeeding. So both lower maternal education and lower rate of exclusive breastfeeding may be the risk factor for the breast abscess diameter > 5 cm during lactation, and the possible reasons are the relative lack of breastfeeding knowledge and lack of assistance for mothers with low education level leading to the low rates of successful exclusive breastfeeding and more breastfeeding breast problems.

At present, there is no sufficient medical evidence to support the timing of antibiotic use, the antibiotic type selection, and the duration of antibiotic using for lactation mastitis. A study [15] has found that antibiotic using does not reduce the frequency of punctures during the treatment of the lactating breast abscesses. Other studies [16] have confirmed that the evidence is insufficient for antibiotic using during the treatment of the lactating breast abscesses. ABM [3] has also pointed out that the primary treatment for lactational mastitis is removing the infected milk in the breast fully by breastfeeding, and both antibiotics used and probiotics used are all the B-level recommendation. This study found that the antibiotics used during lactational mastitis increases the probability of the larger breast abscess (diameter > 5 cm). It further indicates that antibiotics cannot reduce the severity of mammary abscess. RNA technology revealed that healthy milk contains a large number of microorganisms. When mastitis occurs, the species and the quantities of probiotics in the breast decrease, while the quantities of pathogenic bacteria increase [17]. Therefore, the clinical studies have confirmed that some lactobacillus strains extracted from healthy breast milk used for treating mastitis can increase the cure rate with lower rates of recurrence, complication, and delactation when compared with the antibiotic used [18, 19].

This study also found that non-medical breast massage was an independent influencing factor for breast abscess with a maximum diameter > 5 cm during lactation. A large-scale research on the risk factors of mastitis during lactation in China [20] had confirmed that non-medical breast massage increases the incidence of mastitis. The utilization rate of the non-medical breast massage of the lactating postpartum women is 21.8–36% in China [21], which indicated that a great many of the postpartum women suffering from lactation difficulties. The high proportion of the postpartum women in China believed that breast massage for lactogenesis was effictiveness and harmless. In fact, galactostasis and engorgement will increase the pressure in the breast, resulting in milk leakage into the surrounding breast [1]. The breast deep massage may push broken the swollen milk ducts. In some cases of non-bacteremia with galactostasis, a large breast abscess cavity was formed after deep massage, and the contents were leaking milk. Repeated aspiration did not reduce the size, indicating the existence of this problem. The complications of mastitis are mainly caused by obstructed milk drainage. ABM [3] is also clear that deep massage increased inflammation, tissue edema, and microvascular injury. A systematic review [22] concluded that although breast massage may reduce pain, it should not be recommended as the standard of care because it requires extensive training to master atraumatic approach. A new study also confirmed that breast massage did not reverse the development of breast abscesses [23].

Flat or inverted nipples of the breastfeeding women are the independent influencing factors for their maximum diameter of the breast abscess cavity > 5 cm during lactation. Continued lactation during mastitis is an important treatment of the breastfeeding women with flat or inverted nipples [3]. Multiple studies have confirmed that flat or inverted nipples are the risk factors for lactational mastitis [1]. Due to tissue edema during mastitis, the inverted or flat nipples of the women will further increase their pain of sucking milk by babies, aggravate the breast milk flow restriction, and enlarge the size of the breast abscess cavity during lactation. So it is particularly important to give more lactation guidance for the breastfeeding women with flat or inverted nipples.

In this study, there was no significant correlation between the age, the parity, the delivery mode, whether maternal and infant separation of more than 12 h in the past 2 weeks of the patients and their abscess breast abscess size during lactation, which is also found by the study [24]. Although provision of care to breastfeeding women at risk for or affected by mastitis is currently constrained due to a critical lack of high-quality epidemiological evidence about its incidence and risk factors [25]. And there is a lack of randomized-controlled trials focused on the treatment of breast infections. This has resulted in an absence of clinical practice guidelines for the management of breast abscesses and variable practice patterns [26]. A substantial portion of the severity of breast abscess during lactation might be preventable.

## Conclusion

This study is a retrospective, national, multicenter survey with large sample of breast abscesses during lactation, and the surgeons and nurses who participated in this study have been given the standardized training before the study began, so this study may provide medical evidences for the management of breast abscesses during lactation: low education, non-exclusive breastfeeding, fever > 37.5 ℃, inverted or flat nipples, antibiotic used, and non-medical massage history have adverse effects on the severity of breast abscess during lactation, especially for those breast abscesses with the diameter > 5 cm.

## Data Availability

The datasets generated during and/or analyzed during the current study are available from the corresponding author on reasonable request.
